# Strategies for Coping with Occupational Trauma: A Scoping Review of the Police Officer Context

**DOI:** 10.3390/ijerph21070921

**Published:** 2024-07-15

**Authors:** Mantji Juliah Modula, Ellen Mokgobola Mathapo-Thobakgale, Champion N. Nyoni, Ronelle Jansen

**Affiliations:** 1 School of Nursing, Faculty of Health Sciences, University of Free State, Bloemfontein 9300, South Africa; 2 Department of Nursing, Faculty of Health Sciences, University of Limpopo, Polokwane 0727, South Africa

**Keywords:** coping, strategies, police, mental health, law enforcement, occupation, support, trauma

## Abstract

Background: Occupational trauma is heightened among police officers due to their exposure to physical, biological, chemical, and psychological hazards. Sustained occupational trauma results in mental illness among members of the police, which is a public health issue of concern. This study aimed to report a scoping review of the literature on strategies employed by police officers for coping with occupational trauma around the globe. Methods: A search string, formulated from the review question of what is known about the strategies of police officers for coping with occupational trauma, was used to search for articles from databases. A total of 588 hits were screened against inclusion criteria, resulting in 36 full-text studies between 1983 and 2022 being included in this review. Data were extracted using a standardised data extraction tool. The multi-step process was used to analyse the extracted data, integrating quantitative and qualitative approaches. Results: From this review, ‘adaptive coping mechanisms’, involving confrontation; ‘maladaptive coping mechanisms’, such as self-isolation, distancing and substance use; ‘resilience’, relating to mental preparation, and ‘seeking support systems’ from family, colleagues and professionals reflected the strategies used by police officers to cope with occupational trauma. Social stigma related to mental health disorders impacts the strategies used by police officers to cope with occupational trauma. Conclusions: the police management and healthcare practitioners must collaborate towards providing constructive environments that support and strengthen police officers’ strategies for coping with occupational trauma.

## 1. Introduction

Although police officers are the gatekeepers of the criminal justice system and are mandated to maintain and ensure community safety [1], they are also often exposed to occupational trauma. They are usually the first responders to victims and crime scenes [2] and are expected to attend to violent situations [3], along with motor vehicle accidents, child abuse, and domestic violence [4]. Exposure to traumatic events and situations has a detrimental effect on police officers [5], resulting in occupational trauma [6,7] being experienced to a higher degree than most other professions [1].

The obligations and tasks of police officers regarding community safety include mediating conflict situations, working under pressure with limited resources, and exposure to traumatic environments [8], including experiencing colleagues being killed in the line of duty. Substance Abuse and Mental Health Services Administrations [9] further confirm that the police are in danger of being exposed to potentially traumatic situations that may hurt not only themselves but also those in their care. Violanti et al. [10] used the 60-item Spielberger Police Trauma Scale to reveal that police occupational trauma is mostly related to administrative and organisational pressure, physical and psychological threats, and a lack of support. More recently, a study argued that police officers who focused on public protection had increased levels of burnout, compassion fatigue, and secondary trauma during and after the COVID-19 pandemic [11], thus predisposing them to mental health problems. Continuous exposure to occupational trauma increases police officers’ vulnerability to mental health conditions, such as depression, general anxiety disorder (GAD), post-traumatic stress disorder (PTSD), and substance use disorder [6,12,13].

Globally, the prevalence of mental health problems among police officers with occupational stress is estimated at 14.6% for depression, 14.2% for PTSD, 9.6% for GAD, 8.5% for suicidal ideation, 5% for alcohol dependence, and 27% for hazardous drinking [14]. Occupational trauma further increases their risk for PTSD, depression, and alcohol abuse, affecting their physical and mental health [15,16]. Police were found to be at higher suicidal risk related to the effects of occupational trauma than the general population [17]. The high prevalence of physical and mental health disorders among police officers suggests that they employ poor and maladaptive ways of coping with occupational demands. Good adaptation to occupational trauma is associated with active strategies, rather than passive or avoidant strategies, such as alcohol abuse, which are considered maladaptive and negative behaviours [18]. Studies are investigating the impact of negative coping behaviours, such as substance abuse, smoking, an unhealthy diet, and poor sleep, on the workplace stressors contributing to mental health disorders [19].

Coping is derived from a person’s behavioural and cognitive ability to manage internal and external environmental stressors [20]. In essence, coping behaviours, skills and strategies, defence mechanisms, environmental resources, and cognitive ability assist individuals in managing emotional, physical, and social stressors [21]. Strategies to react to a stressor can either be effective or ineffective [22]. Coping strategies are mechanisms that individuals can apply to influence their psychological well-being after experiencing a critical incident [20]. Notably, the context in which a person’s coping strategy is applied influences the outcome of the coping strategy. The literature reports several strategies that are used by police officers to cope with occupational trauma. However, several studies found that problem-focused coping strategies were influential in the policing context [2], where police adapted to stressful incidents or situations [22]. 

Although research on coping strategies of police officers experiencing stress has been conducted, it lacks a clear indication of the existing police coping strategies of this population in lower-middle-income countries (LMIC), where there is often a lack of adequate resources for support. The researchers also found a lack of guidance for treating occupational trauma in policing in LMICs [23]. There is a gap in the literature regarding evidence of the coping strategies used by police officers to cope with occupational trauma, and how it impacts their mental health. This scoping review reports on the strategies for coping with occupational trauma by members of the police around the globe, as there is a lack of literature about LMICs. Insights into reported coping strategies would be useful to managers in developing collaborative efforts and models to actively support police officers in dealing with occupational trauma. 

## 2. Materials and Methods

### 2.1. Research Design

This review sought to answer the question: “What is known about the strategies of police officers for coping with occupational trauma?” The methodological framework delineated by Peters et al. [24] was adhered to while conducting the scoping review. These procedural steps encompassed formulating a comprehensive search strategy, a literature search, screening and selecting studies, data extraction, and data analysis.

#### 2.1.1. Search Strategy

The search strategy for this review specified the search terms and databases consulted [24]. The search terms were derived from the key concepts within the review question, as mapped using the Population, Concept and Context (PCC) approach. An information specialist from a university library supported this study by refining the search string, in addition to a preliminary “quick and dirty” search. The final search string is presented as:

(Police OR “Police force” OR Policemen OR Policewomen OR “officers of the law” OR “forces of law and order” OR “law enforcement officers” OR “law enforcement agency” OR constabulary) AND (“Coping strategies” OR “Coping Skills” OR “coping mechanisms” OR “Coping resources” OR “Internal resources” OR Self-help OR Self-management) AND (“Occupational trauma” OR “Workplace trauma” OR “trauma in the workplace”) 

#### 2.1.2. Searching the Literature

The search string was applied to databases on the EBSCOhost platform, which was accessed through the university to search for literature. Academic Search Complete, PsycINFO, ERIC, MEDLINE with Full Text, Health Source, Nursing/Academic Edition, SPORTDiscus with Full Text, CINAHL with Full Text, SocINDEX with Full Text, Africa-Wide Information, Communication & Mass Media Complete, Humanities Source, and PsycEXTRA were the databases included in this review. The databases increased the search strategy of locating keywords from titles, abstracts and keywords of articles. 

#### 2.1.3. The Screening and Selection of Literature

The search output, in the form of titles and abstracts, was initially screened for relevance to the study title and review question. This output was captured in research information system (RIS) format and exported and uploaded to the Rayyan platform. This cloud-based password-protected service allowed authors to be blinded during the screening and selection process. One researcher could not view the other researcher’s selection during this process. The four researchers, MJM, EMM-T, RJ, and CNN, independently used the Rayyan platform to either include, exclude, or indicate uncertainty towards the studies based on their titles and abstracts.

The inclusion and exclusion criteria were: 

Inclusion criteria

- Coping strategies related to occupational trauma or workplace trauma
- Police and law enforcement agents
- Published primary research

Exclusion criteria

- Secondary data articles (other scoping and systematic review reports)

**Figure 1:**
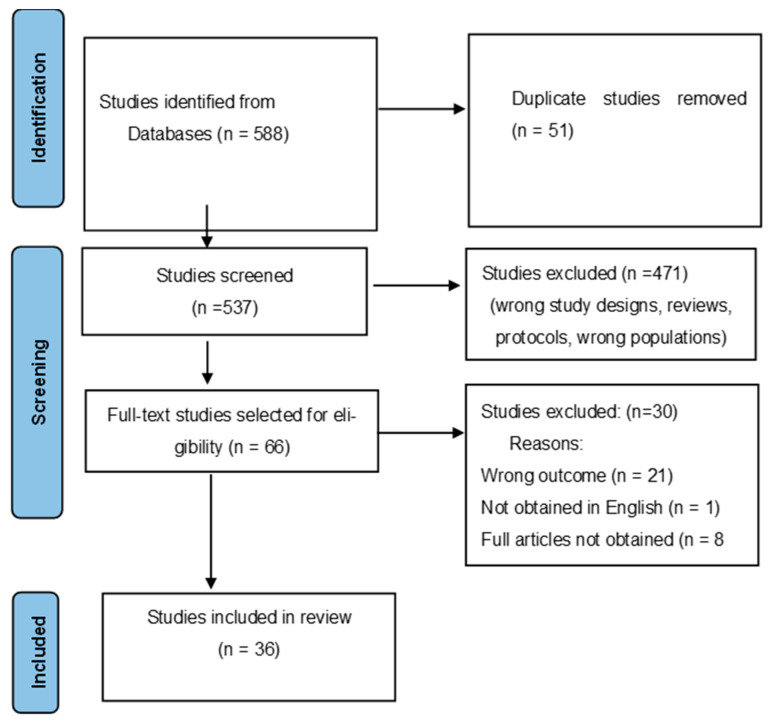
PRISMA Flow diagram of included studies (adapted from Moher, Liberati, Tetzlaff, Altman, and The PRISMA Group (2009)).

The inclusion and exclusion criteria assisted in including the studies related to the coping strategies of police officers regarding occupational trauma. The PRISMA-ScR process of screening and selecting articles was applied to screen the titles and abstracts against the inclusion and exclusion criteria, eliminating those not meeting the inclusion criteria. The researchers discussed the selection and decided to include studies based on the eligibility criteria. Full-text articles of the included titles and abstracts were sought from the university library. The authors used the same inclusion and exclusion criteria in screening the full-text articles (see Figure 1: PRISMA-ScR Diagram). The initial search identified a total of 588 records from the databases. Fifty-one duplicate studies were removed, 471 were excluded based on eligibility criteria, 66 full texts were selected, and 30 of them were excluded due to wrong outcome, not available and not in the English language, as illustrated in Figure 1 below.

### 2.2. Data Extraction

The four authors piloted an author-generated extraction tool and used this to extract data from the included 36 studies. These studies were equally distributed among the four authors for data extraction using the Google Forms platform. The data extraction focused on the characteristics of the articles, including the authors, year of publication, title, country where the research was conducted, the research purpose, design, population and sampling, data collection methods, and data analysis techniques. Furthermore, data to answer the specific review question were also extracted, such as the specific coping strategy reported, the results of using such a strategy, and any recommendations from the researchers. The data extracted by four researchers independently had very few discrepancies, thus maintaining the intercoder reliability, as the data were in consensus with each other. 

### 2.3. Data Analysis

The data analysis integrated qualitative and quantitative approaches [24]. The characteristics were analysed through descriptive statistics based on frequencies and measures of central tendency, while the concept and outcomes underwent multiple phases of inductive qualitative analysis. Using the Atlas Ti version 9 software program, data was coded through initial and/or open coding. Codes were clustered based on similarities influenced by the review question. Patten coding was applied to identify patterns between the clusters and link these to the information found in the literature. The authors reached a consensus on the themes and subthemes that emerged from the analysis, which validated the intercoder reliability and consistency.

## 3. Results

The results reflect the characteristics of the included articles and themes that mention coping strategies.

### 3.1. Article Characteristics

The review included 36 English language articles between 1983 and 2022. The publication year and frequencies were 2022 (*n* = 6), 2020 (*n* = 6), 1983–2000 (*n* = 6), 2018 (*n* = 4), 2017 (*n* = 4), 2021 (*n* = 3), 2016 (*n* = 2), 2011 (*n* = 2), 2014 (*n* = 1), 2015 (*n* = 1), and 2019 (*n* = 1). The countries of publication and the study frequencies (*n*) are indicated as follows: United States of America (USA) (*n* = 14), Canada (*n* = 2), Italy (*n* = 2), Germany (*n* = 2), United Kingdom (*n* = 2), Portugal (*n* = 2), South Africa (RSA) (*n* = 2), and (*n* = 1) from each of the following: Australia, Brazil, Finland, India, Bulgaria, The Netherlands, New Zealand, Slovakia, Republic of Korea, and Sweden.

The methodological research designs utilised by the selected studies include quantitative (*n* = 21), qualitative (*n* = 13), and mixed methods (*n* = 2) designs. The study population included police members, including police personnel, law enforcement, police officers, public safety personnel, policemen, police recruits, police educators, crime scene police investigators, and police patrol personnel.

### 3.2. Coping Strategies of Police Officers

A summary of the characteristics of the included articles analysed through an inductive process addressed the question of what is known about the coping strategies of police. The data analysis generated four themes related to the coping strategies of police officers, as indicated in Table 1 below. 

#### 3.2.1. Adaptive Coping Strategies (Theme 1)

Adaptive coping strategies encompass a variety of approaches that the police use to effectively manage their occupational trauma. Under this theme, confronting coping was reported by [25,26,27], as it focused on directly addressing occupational trauma. Emotion-focused coping, on the other hand, focused on regulating emotional responses to occupational trauma [28]. Positive reappraisal and reframing involved an internal process of viewing occupational trauma in a positive light in an attempt to alleviate its impact [18,29]. Problem-focused coping involves a strategy of actively seeking solutions to the stressor [30,31]. Task-orientated coping, identified by three studies, entails organising and prioritising tasks to manage stressors effectively [31,32,33]. Spirituality and religion have also been recognised as coping strategies [34,35]. Finally, training in stress management techniques, as highlighted, involved equipping individuals with the skills to effectively manage and reduce stress levels [36,37,38,39]. In addition, this review highlights that police officers undergo training sessions and receive education from professionals on the causes and signs of occupational trauma, as well as stress management techniques (mindfulness, exercises, and relaxation) to maintain good mental health. These reported coping strategies reflected an adaption of the individual to the occupational trauma.

#### 3.2.2. Maladaptive Coping Strategies (Theme 2)

Maladaptive coping mechanisms were reported as a spectrum of strategies that the police used when faced with occupational trauma, and which often exacerbate rather than alleviate the effects of such trauma. In most cases, such maladaptive coping strategies resulted in more harm to the police officers or their immediate families. Distancing, as a maladaptive strategy, involves creating emotional or physical distance from occupational trauma [40,41]. Similarly, self-distraction, as highlighted, entails temporarily diverting attention away from the effects of occupational trauma with the hope that ‘it will all go away’ [18]. Escape avoidance, recognised by the studies, involves evading the effect of occupational trauma altogether [33]. Social isolation implies withdrawing from social connections during and after the times related to occupational trauma [36,40]. Self-blame, denial, and repression strategies involve attributing fault to oneself, refusing to acknowledge the occupational trauma, or suppressing any associated emotions [18,20]. The findings indicate self-distancing is more common in males, while denial and humor were more common in female police officers. Mental disengagement, recognised by studies, encompasses mentally detaching from the effect of the trauma or its consequences [31,41]. Substance use, as highlighted, mentioned turning to drugs or alcohol as a means of coping with occupational trauma, often leading to further complications, such as alcoholism [42,43,44]. Maladaptive coping strategies underscore unhealthy responses by the police related to occupational trauma.

#### 3.2.3. Resilience Coping (Theme 3) 

The resilience coping theme reflected on strategies that emphasise the ability of the police to adapt and bounce back from occupational trauma. It encompasses a range of coping strategies. Among these strategies, humour emerged as a potent tool, as evidenced in this review [45]. Studies highlight the role of laughter in promoting resilience among the police faced with occupational trauma. Visualisation and self-mental preparation enable individuals to mentally rehearse and prepare for the potential stressors associated with occupational trauma [46,47]. Self-emotion regulation, self-control, self-responsibility, and acceptance empower individuals to regulate their emotions, exercise discipline, and take ownership of their circumstances, thereby fostering resilience. Similarly, self-reliance, planning, and knowledge expansion, as highlighted by studies, equip the police with the necessary tools and resources to address challenges and adapt to change proactively [43,48]. Venting their experiences or emotions provides an outlet for the police to express and process their feelings, promoting resilience through emotional release and catharsis [49,50,51]. These diverse coping strategies collectively contributed to enhancing resilience, enabling the police to thrive in the face of occupational trauma. However, the review reveals that some police officers accepted their own vulnerability from exposure to traumatic events and were willing to seek assistance from available support systems.

#### 3.2.4. Seeking Support Systems (Theme 4)

The seeking of support systems by the police is integral to effectively navigating the effects of occupational trauma, and various strategies were clustered within the theme of ‘seeking support systems’. Social support emerges as a cornerstone of the seeking of support systems by the police, with numerous studies underscoring its significance. The results highlight the pivotal role of social networks in providing tangible and emotional support during and after experiencing occupational trauma [52,53,54]. Moreover, emotional and psychological support was reported as a crucial strategy in bolstering resilience and being part of the support system, underscoring the importance of empathetic listening and validation in promoting well-being [55,56]. Additionally, as stressed, professional support plays a vital role in addressing complex challenges, emphasising the expertise and resources that are part of the social support systems available to police while facing the effects of occupational trauma [57]. The review found police officers seek social support in sharing the pressure of occupational trauma experienced in their line of duty. In addition, some seek emotional support from their team leaders, even though there is evidence of mistrust in them. These findings underscore the multifaceted nature of support systems and highlight the importance of nurturing diverse networks to foster resilience and well-being among members of the police.

## 4. Discussion

This paper sought to report on a scoping review of the literature on the strategies employed by police officers for coping with occupational trauma. The review underscores that the mental health challenges of police officers are a public health issue by unveiling their reported strategies to cope with occupational trauma [58]. The findings highlight that police officers adopted strategies such as adaptive coping mechanisms, maladaptive coping mechanisms, resilience, and seeking support systems to deal with occupational trauma. These strategies can be used to suggest approaches to support the police undergoing occupational trauma in various settings.

Most of the studies included in this review were conducted in the United States. The United States faces a significant challenge regarding violence and gun control, and the involvement of police officers in such situations is evident [59]. In addition, the United States has reported significant investments in the care and well-being of its police workforce, which may include resources for conducting and publishing research on the welfare of the police [60]. It would be helpful to strengthen the literature by investigating the strategies used to cope with occupational trauma in non-English speaking settings or settings with a different social order, such as the Middle and Far East.

As established, police officers are continuously exposed to potentially traumatic events in the line of duty, which can have profound effects on their mental well-being [8]. Understanding how they cope with these challenges and the support systems available to them is essential for promoting their psychological health. The findings of this review indicate that police officers utilise various coping mechanisms to manage their professional stress and its emotional toll. Adaptive coping strategies, such as confrontation, problem-solving, positive reappraisal, spirituality, task orientation, and stress management techniques, have been identified as beneficial for maintaining psychological well-being, a finding supported by Kukić et al. [61]. The findings show that coping strategies enable officers to directly address their challenges, regulate their emotions, and maintain a positive outlook in the face of adversity. The previous research supports the importance of experience and training programmes in developing practical coping skills [1], which indicates that experienced police officers with broad expertise adapt better to occupational trauma than novices [62]. Hence, police officers use this practice to express their emotions and seek peer support, which can help alleviate stress and foster a sense of camaraderie.

Conversely, maladaptive coping mechanisms in this study are associated with adverse outcomes, such as stress, depression, and even suicide among police officers. These are often employed as a means of avoiding or numbing the effects of occupational trauma [60]. In support of this finding, a previous review study with a similar population found that high levels of avoidance have been linked to suicide ideation and self-harm, highlighting the severe consequences of ineffective coping strategies [1]. Substance use among police officers, particularly alcohol, is used to cope with the stress and trauma of the profession [63]. The results indicate mental disengagement as a maladaptive coping strategy to deal with occupational trauma. Comparably, the previous review’s results found that disengagement strategy efforts related to adjustment [64] 

Resilience coping strategies are reported in this study as crucial in supporting police officers in engaging with their stressful situations and maintaining their psychological well-being. Resilience coping strategies, such as humour, self-regulation, self-reliance, self-control, planning and knowledge expansion, enable officers to adapt to the challenging circumstances brought about by occupational trauma and to remain effective in their roles [65]. Comparably, research suggests that resilience can mediate the relationship between social support and mental disorders, indicating its importance in promoting psychological health [66]. 

The effectiveness of coping strategies is influenced by organisational factors, such as workplace authority and access to decision-making [10]. Wasserman et al. [2] reported that the effectiveness of coping strategies depends on the organisation’s support systems and resources, with greater autonomy and involvement in decision-making leading to more effective coping. Support systems are essential for assisting police officers in coping with occupational trauma and maintaining their mental well-being. Social support networks, including colleagues, family and friends, provide emotional assistance and comfort to officers during difficult times [67]. Professional support from psychologists, counsellors, and mental health therapy programmes is also crucial in addressing the psychological impact of occupational trauma and helping officers develop effective coping strategies [57]. Comparably, a conducted review indicates that the modification of occupational demands enhances coping skills and further reduces burnout among police officers [68]. The findings of this review showed that police officers preferred receiving professional support from outside the workplace to deal with trauma. However, the organisational cultures and stigmas surrounding mental health may deter police officers from seeking professional support within the workplace, highlighting the need for efforts to reduce stigma and promote access to psychological support services in a culturally appropriate approach [1]. Hence, the effective promotion and continuity of mental health interventions require strong organisational support, with the responsibility shared by police officers and their supervisors [63].

In re-engineering the mental health support for police officers faced with occupational trauma, we recommend:

- Amplifying the awareness of the available support and social programmes that target police officers, both immediately after facing occupational trauma and in the long term;
- The development and implementation of policies in the workplace to support the mental health programmes of police officers;
- Collaboration between police administration and healthcare service providers in developing tailor-made and contextually relevant care models that are focused on police mental health;
- Investing in research activities that report on the impact of coping strategies on specific health outcomes within the police workforce;
- Future research to support the establishment of mental health programmes to equip police officers with cognitive therapies to enhance their coping skills to deal with occupational trauma.

The study’s limitations are, firstly, that the study’s purpose was to review the literature on what is known about the coping strategies used by police officers regarding occupational trauma, but the search could find fewer publications from LMICs. Secondly, police officers have different ranking positions with different levels of experience. However, this indicated a lack of information in the literature on the coping strategies used by all police officers. Thirdly, the search strategy did not include coping skills, which would have identified more publications regarding coping strategies.

## 5. Conclusions

The mental health of police officers is a public health matter of concern, and comprehensive contextually relevant models to address their mental health must be developed as a matter of urgency. This study focused on describing reported strategies that police officers use to cope with occupational trauma, which includes adaptive and maladaptive strategies, resilience approaches, and social support systems. As countries re-imagine their approaches towards being on track with the United Nations’ sustainable development goals (SDGs), the mental health of police officers must be prioritised. This review of strategies could provide a point of departure to guide the development of approaches to address the occupational stress of police officers.

## Figures and Tables

**Table 1:** Coping strategies of police officers.

Themes	Subthemes	References	Number of References and Countries (*n*)
Adaptive coping strategies	Confrontive coping (directly addressing and tackling occupational trauma head-on)	Kopel and Blackman (1996); Violanti et al. (1992); Rodrigues and Ribeiro (2016)	(*n* = 3) RSA = 1 USA = 1 Brazil = 1
	Emotion-focused coping (focused on regulating emotional responses to trauma)	Patterson (1999); Williams (2016); Sollie et al. (2017); Rodrigues and Ribeiro (2016); Queirós et al. (2020)	(*n* = 5) USA = 2 Brazil = 1 Portugal = 1 Netherlands = 1
	Positive reappraisal (internal process of viewing trauma in a more positive light to alleviate their impact)	Kopel and Blackman (1996); Violanti et al. (1992); Bonner and Brimhall (2022)	(*n* = 3) SA = 1 USA = 2
	Positive reframing (the process of viewing trauma in more positive light, alleviating the impact)	Arble et al. (2017); Acquadro Maran et al. (2015)	(*n* = 2) Italy = 1 USA = 1
	Problem-focused solving coping (actively seeking solutions to stressors)	Leonard and Alison (1999); Kopel and Blackman (1996); Patterson (1999); Violanti et al. (1992); Rodrigues and Ribeiro (2016); Ryu et al. (2020); Furmeen and Reddy (2019)	(*n* = 7) Brazil = 1 India = 1 RSA = 1 South Korea = 1 USA = 2
	Task-orientated (organising and prioritizing tasks to manage stressors effectively)	Queirós et al. (2020); Rodrigues and Ribeiro (2016); Carrier (2020)	(*n* = 3) Brazil = 1 Portugal = 1 USA = 1
	Spirituality/religion (emotional support through spiritual connections and religious belief)	Bonner and Brimhall (2022); Hartman (2021); Acquadro Maran et al. (2015); Furmeen and Reddy (2019)	(*n* = 4) India = 1 Italy = 1 USA = 2
	Stress management techniques training (equipping individuals with skills to reduce and manage stress levels effectively)	Manzella and Papazoglou (2014); Acquadro Maran et al. (2018); Seigfried-Spellar, (2018); Sadulski (2017); Kaur et al. (2021); Denk-Florea et al. (2020)	(*n* = 7) Bulgaria = 1 England = 1 Germany = 1 Italy = 1 USA = 3
Maladaptive coping strategies	Distancing (creating emotional or physical distance from trauma)	Furmeen and Reddy (2019); Seigfried-Spellar (2018); Sollie et al. (2017); Kopel and Blackman (1996); Violanti et al. (1992); Civilotti et al. (2021); Backteman-Erlanson et al. (2013)	(*n* = 6) India = 1 Italy = 1 Netherlands = 1 RSA = 1 Sweden = 1 USA = 1
	Self-distraction (diverting attention away from the effects of trauma temporarily)	Acquadro Maran et al. (2015); Seigfried-Spellar (2018)	(*n* = 2) Italy = 1 USA = 1
	Escape/avoidance (evading the effect of trauma altogether)	Carrier (2020); Kopel and Blackman (1996); Violanti et al. (1992); Acquadro Maran et al. (2018); Rodrigues and Ribeiro (2016); Ryu et al. (2020)	(*n* = 6) Brazil = 1 Italy = 1 RSA = 1 South Korea = 1 USA = 2
	Social isolation (withdrawing from social connections during and after the times related to trauma)	Manzella and Papazoglou (2014); Furmeen and Reddy (2019)	(*n* = 2) Bulgaria = 1 India = 1
	Self-blame/denial/repression (attributing fault to oneself, refusing to acknowledge trauma, or suppressing any associated emotions)	Acquadro Maran et al. (2015); Civilotti et al. (2021)	(*n* = 2) Italy = 2
	Mental disengagement (mentally detaching from effects of trauma or its consequences)	Leonard and Alison (1999); Seigfried-Spellar, (2018); Violanti et al. (1983); Acquadro Maran et al. (2015); Civilotti et al. (2021)	(*n* = 5) Italy = 2 UK = 1 USA = 2
	Substance use (turning to drugs or alcohol to cope with the trauma)	Leino et al. (2011); Powell et al. (2014); Manzella and Papazoglou (2014); Faulkner (2018); Leonard and Alison (1999); Seigfried-Spellar, (2018); Violanti et al. (1983); Acquadro Maran et al. (2015); Furmeen and Reddy (2019)	(*n* = 9) Australia = 1 Bulgaria = 1 Canada = 1 Finland = 1 India = 1 Italy = 1 USA = 2 UK = 1
Resilience coping	Humour (highlights the role of laughter in promoting resilience among those faced with trauma)	Denk-Florea et al. (2020); Arble et al. (2017); Acquadro Maran et al. (2015)	(*n* = 3) Italy = 1 UK = 1 USA = 1
	Visualisation/self-mental preparation (enables individuals to mentally rehearse and prepare for potential stressors associated with trauma)	Ondrejková and Halamová (2022); Denk-Florea et al. (2020); Civilotti et al. (2021); Sollie et al. (2017)	(*n* = 4) England = 1 Italy = 1 Netherlands = 1 Slovakia = 1
	Self-emotion regulation (empowers individuals to regulate their emotions)	Kopel and Blackman (1996); Faulkner (2018); Ondrejková and Halamová (2022)	(*n* = 3) Canada = 1 RSA = 1 Slovakia = 1
	Self-control (empowers individuals to exercise discipline)	Kopel and Blackman (1996); Violanti et al. (1992); Ondrejková and Halamová (2022)	(*n* = 3) RSA = 1 Slovakia = 1 USA = 1
	Self-responsibility/acceptance (empowers individuals to take ownership of their circumstances)	Kopel and Blackman (1996); Violanti et al. (1992); Acquadro Maran et al. (2015); Rodrigues and Ribeiro (2016); Furmeen and Reddy (2019)	(*n* = 5) Brazil = 1 India = 1 Italy = 1 RSA = 1
	Self-responsibility/acceptance (empowers individuals to take ownership of their circumstances)	Kopel and Blackman (1996); Violanti et al. (1992); Acquadro Maran et al. (2015); Rodrigues and Ribeiro (2016); Furmeen and Reddy (2019)	(*n* = 5) Brazil = 1 India = 1 Italy = 1 RSA = 1
	Self-reliance (equips people with the tools and resources necessary to proactively address challenges and adapt to change)	Williams (2016); Papazoglou and Andersen (2014); Backteman-Erlanson et al. (2011); Queirós et al. (2020)	(*n* = 3) Portugal = 1 Sweden = 1 USA = 1
	Planning (proper time management for work-life balance)	Bonner et al. (2022); Acquadro Maran et al. (2015)	(*n* = 2) Italy = 1 USA = 1
	Knowledge expansion (acquiring more information about traumatic events, and focusing on self-development)	Ondrejková (2022); Backteman-Erlanson et al. (2011); Steyn and Klopper (2020)	(*n* = 3) Slovakia = 1 Sweden = 1 RSA = 1
	Venting the experiences or emotions (sharing and expressing their challenges)	Leonard et al. (1999); Stephens et al (2000); Bonner et al. (2022); Acquadro Maran et al. (2015)	(*n* = 4) Italy = 1 New Zealand = 1 USA = 1 UK = 1
Seeking support systems	Social support (highlights the role of social networks in providing emotional support during and after experiencing trauma)	Kaur et al. (2021); Carrier (2020); Kopel and Blackman (1996); Casas (2021); Sadulski (2017); Williams (2016); Anderson et al. (2022); Violanti et al. (1992); Leonard and Alison (1999); Bonner and Brimhall (2022); Sollie et al. (2017); Seigfried-Spellar (2018); Backteman-Erlanson et al (2011); Backteman-Erlanson et al. (2011); Acquadro Maran et al. (2015); Steyn and Klopper (2020)	(*n* = 17) Canada = 1 India = 1 Italy = 2 Netherlands = 1 RSA = 1 Sweden = 3 UK = 1 USA = 8
	Emotional and psychological support (emphasises the importance of empathetic listening and validation in promoting well-being)	Hartman (2021); Beahm (2022); Bonner and Brimhall (2022); Sollie et al. (2017); Acquadro Maran et al. (2015); Papazoglou et al. (2018); Ondrejková and Halamová (2022); Turner-Barnes (2017); Leino et al. (2011)	Italy = 1 Netherlands = 1 Slovakia = 1 USA = 3
	Professional support (the expertise and resources that are part of support systems available for people undergoing the effects of trauma)	Kaur et al. (2021); Steyn and Klopper (2020); Anderson et al. (2022); Casas (2021)	(*n* = 4) Canada = 1 India = 1 RSA =1 USA = 1

## Data Availability

The data presented in this study are available upon request from the corresponding author, M.J.M.
